# *trans*-Carbonyl­chloridobis[tris­(4-meth­oxy­phen­yl)phosphane-κ*P*]rhodium(I)

**DOI:** 10.1107/S1600536811046150

**Published:** 2011-11-05

**Authors:** Stefan Warsink, Andreas Roodt

**Affiliations:** a Department of Chemistry, University of the Free State, PO Box 339, Bloemfontein 9300, South Africa

## Abstract

The title complex, [RhCl(C_21_H_21_O_3_P)_2_(CO)], is a rhodium analogue to Vaska’s complex with *para*-meth­oxy substituents on the six phosphan­yl–aryl units. Two independent mol­ecules are present in the unit cell, with their metal atoms both located on an inversion centre. This causes the chloride and carbonyl ligands to exhibit a positional disorder in a 0.5:0.5 ratio. The two Rh^I^ atoms exhibit a distorted square-planar geometry. There are a few weak intra­molecular C—H⋯*X* inter­actions (*X* = O, Cl). Inter­estingly, no significant inter­molecular inter­actions are found between the two independent mol­ecules.

## Related literature

For background to Vaska’s complex, see: Angoletta (1959 ▶); Vaska & Di Luzio (1961 ▶). For related literature on rhodium Vaska complexes, see: Basson *et al.* (1990 ▶); Clarke *et al.* (2002 ▶); Kemp *et al.* (1995 ▶); Rheingold & Geib (1987 ▶); Roodt *et al.* (2003 ▶); Wilson *et al.* (2002 ▶). For similar complexes, see: Burgoyne *et al.* (2010 ▶), Meijboom *et al.* (2006 ▶), Monge *et al.* (1983 ▶); Otto *et al.* (1999 ▶). Synthetic details are given in McCleverty & Wilkinson (1990 ▶).
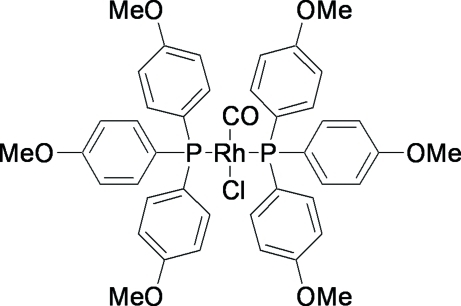


## Experimental

### 

#### Crystal data

[RhCl(C_21_H_21_O_3_P)_2_(CO)]
*M* *_r_* = 871.07
Triclinic, 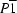

*a* = 7.8350 (4) Å
*b* = 12.3151 (8) Å
*c* = 21.0591 (13) Å
α = 90.995 (2)°
β = 99.591 (2)°
γ = 101.220 (2)°
*V* = 1962.7 (2) Å^3^
*Z* = 2
Mo *K*α radiation
μ = 0.64 mm^−1^
*T* = 100 K
0.24 × 0.16 × 0.10 mm

#### Data collection

Bruker APEXII CCD diffractometer
Absorption correction: multi-scan (*SADABS*; Bruker, 2004 ▶) *T*_min_ = 0.887, *T*_max_ = 0.937
26016 measured reflections
9327 independent reflections
6401 reflections with *I* > 2σ(*I*)
*R*_int_ = 0.098

#### Refinement

*R*[*F* ^2^ > 2σ(*F* ^2^)] = 0.052
*wR*(*F* ^2^) = 0.144
*S* = 1.04
9327 reflections
517 parameters
H-atom parameters constrained
Δρ_max_ = 2.45 e Å^−3^
Δρ_min_ = −1.17 e Å^−3^

### 

Data collection: *APEX2* (Bruker, 2005 ▶); cell refinement: *SAINT-Plus* (Bruker, 2004 ▶); data reduction: *SAINT-Plus* and *XPREP* (Bruker, 2004 ▶); program(s) used to solve structure: *SHELXS97* (Sheldrick, 2008 ▶); program(s) used to refine structure: *SHELXL97* (Sheldrick, 2008 ▶); molecular graphics: *Mercury* (Macrae *et al.*, 2008 ▶); software used to prepare material for publication: *WinGX* (Farrugia, 1999 ▶).

## Supplementary Material

Crystal structure: contains datablock(s) global, I. DOI: 10.1107/S1600536811046150/wm2548sup1.cif

Structure factors: contains datablock(s) I. DOI: 10.1107/S1600536811046150/wm2548Isup2.hkl

Additional supplementary materials:  crystallographic information; 3D view; checkCIF report

## Figures and Tables

**Table:** 

Rh1—C1	1.699 (12)
Rh1—P1	2.3257 (10)
Rh1—Cl1	2.416 (5)
Rh2—C23	1.751 (11)
Rh2—P2	2.3321 (11)
Rh2—Cl2	2.410 (4)
C1—O1	1.157 (18)
C23—O5	1.171 (13)

**Table:** 

C1—Rh1—P1	90.5 (4)
P1—Rh1—Cl1	91.20 (9)
C23—Rh2—P2	91.9 (4)
P2—Rh2—Cl2	92.06 (10)

**Table 2:** Hydrogen-bond geometry (Å, °)

*D*—H⋯*A*	*D*—H	H⋯*A*	*D*⋯*A*	*D*—H⋯*A*
C14—H046⋯Cl1	0.95	2.81	3.191 (5)	105
C32—H021⋯Cl2	0.95	2.82	3.157 (5)	102
C8—H04*A*⋯Cl1^i^	0.98	2.73	3.693 (7)	169
C8—H04*A*⋯O1^i^	0.98	2.52	3.486 (18)	168

Symmetry code: (i) 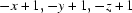.

**Fig. 1.**
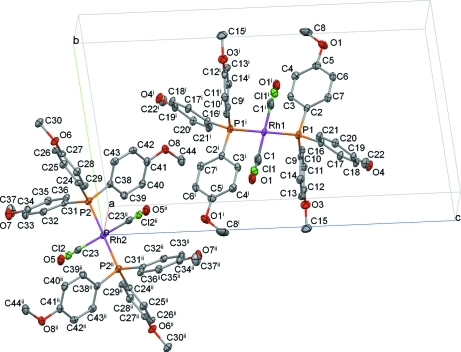
Molecular structure of (I), showing the unit cell to clarify the special positions of the two rhodium atoms. Displacement ellipsoids are drawn at the 50% probability level. H-atoms have been omitted for clarity [Symmetry code: (i) -x, 1 - y, 1 - z; (ii) -x, -y, -z].

## supplementary crystallographic information

### Comment

Vaska's complex was first synthesized by Angoletta (1959) and later correctly formulated as *trans*-[IrCl(CO)(PPh_3_)_2_] (Vaska & Di Luzio, 1961). This compound has been used in various catalytic processes and it or its analogues are often employed as model compounds (Rheingold & Geib, 1987; Basson *et al.*, 1990; Kemp *et al.*, 1995; Roodt *et al.*, 2003).

Various 'Vaska complexes' have been synthesized, exploring different metals but especially introducing different substituents on the phosphane ligands. These modifications have an impact on the steric hindrance around the metal (Clarke *et al.*, 2002; Wilson *et al.*, 2002), but in the case of *para*-substituted triaryl phosphanes the effect is purely electronic (Monge *et al.*, 1983; Otto *et al.*, 1999; Meijboom *et al.*, 2006; Burgoyne *et al.*, 2010). Since only limited data are available on this kind of complexes, we have prepared the rhodium analogue (I), [RhCl(C_21_H_21_O_3_P)_2_(CO)], bearing relatively electron-rich tri(*para*-methoxyphenyl)-phosphane ligands.

Two independent half-molecules are present in the asymmetric unit of compound (I), in each case with the Rh^I^ atoms located on inversion centres. The metal atoms display a distorted square planar geometry with the phosphane ligands located in mutual *trans*-positions (Fig. 1). Selected bond lenghts and angles are presented in Table 1.

The carbonyl moiety has a slightly bent geometry, with Rh—C—O angles of 173.2 (14)° and 176.8 (16)° for the two molecules, respectively. In solution infrared spectroscopy only one signal was observed for the carbonyl ligand at 1974 cm^-1^. Also in solid state infrared spectroscopy of the amorphous material, only one signal was observed at 1964 cm^-1^. Only when a crystalline sample was analysed, two signals were observed at 1956 and 1973 cm^-1^, showing the stretching vibrations of both the independent carbonyl ligands. In ^31^P NMR the signal for the phospine ligands was observed at 24.95 ppm with a *J*_Rh—P_ of 124.5 Hz, which is in line with analogous complexes.

The Rh—P bond lengths fall in the range of other, analogous rhodium Vaska complexes. In contrast, the bonds of the metal to the carbonyl and chlorido ligands are significantly influenced by the electron-donating phosphane ligands. The bond to the chlorido ligand is the longest reported for this kind of complexes bearing triaryl phosphanes. The same influence is also notably present in the bonding of the carbonyl ligand. Its bond to the rhodium atom is quite short, which indicates significant metal-to-ligand electron donation. As a consequence, the C—O bond is lengthened.

There are a few weak intramolecular C—H···*X* interactions (*X* = O, Cl), which are listed in Table 2. Interestingly, no intermolecular interactions are found between the two independent molecules.

### Experimental

Compound (I) was synthesized by slowly adding 4 equivalents of tri(4-methoxyphenyl)phosphane to a dimethyl formamide solution of [RhCl(CO)_2_]_2_ (McCleverty & Wilkinson, 1990). The product was precipitated with ice water and isolated by filtration. Crystallization was performed by dissolving the complex in a small amount of dichloromethane which was then carefully layered with approximately 5 volumetric equivalents of hexane. The mixture was stored in a loosely closed vessel, from which yellow crystals precipitated.

### Refinement

The aromatic and methyl H atoms were placed in geometrically idealized positions (C—H = 0.93–0.98) and constrained to ride on their parent atoms with *U*_iso_(H) = 1.2*U*_eq_(C) for aromatic protons and *U*_iso_(H) = 1.5*U*_eq_(C) for methyl protons. The disordered Cl and CO ligands were constrained to have occupancies of 0.5 at each of the two positions. The highest residual electron density was located 0.90 Å from Rh1 and was essentially meaningless. The deepest hole was located 1.00 Å from Rh1.

### Crystal data

**Table:**

[RhCl(C_21_H_21_O_3_P)_2_(CO)]	*Z* = 2
*M**_r_* = 871.07	*F*(000) = 896
Triclinic, *P*1	*D*_x_ = 1.474 Mg m^−^^3^
Hall symbol: -P 1	Mo *K*α radiation, λ = 0.71073 Å
*a* = 7.8350 (4) Å	Cell parameters from 6500 reflections
*b* = 12.3151 (8) Å	θ = 2.9–28.1°
*c* = 21.0591 (13) Å	µ = 0.64 mm^−^^1^
α = 90.995 (2)°	*T* = 100 K
β = 99.591 (2)°	Cuboid, yellow
γ = 101.220 (2)°	0.24 × 0.16 × 0.10 mm
*V* = 1962.7 (2) Å^3^	

### Data collection

**Table:**

Bruker APEXII CCD diffractometer	9327 independent reflections
Radiation source: sealed tube	6401 reflections with *I* > 2σ(*I*)
graphite	*R*_int_ = 0.098
Detector resolution: 512 pixels mm^-1^	θ_max_ = 28°, θ_min_ = 2.0°
φ and ω scans	*h* = −10→6
Absorption correction: multi-scan (*SADABS*; Bruker, 2004)	*k* = −15→16
*T*_min_ = 0.887, *T*_max_ = 0.937	*l* = −27→27
26016 measured reflections	

### Refinement

**Table:**

Refinement on *F*^2^	Primary atom site location: structure-invariant direct methods
Least-squares matrix: full	Secondary atom site location: difference Fourier map
*R*[*F*^2^ > 2σ(*F*^2^)] = 0.052	Hydrogen site location: inferred from neighbouring sites
*wR*(*F*^2^) = 0.144	H-atom parameters constrained
*S* = 1.04	*w* = 1/[σ^2^(*F*_o_^2^) + (0.0711*P*)^2^] where *P* = (*F*_o_^2^ + 2*F*_c_^2^)/3
9327 reflections	(Δ/σ)_max_ = 0.003
517 parameters	Δρ_max_ = 2.45 e Å^−^^3^
0 restraints	Δρ_min_ = −1.17 e Å^−^^3^

### Special details

**Table:**

Experimental. The intensity data was collected on a Bruker X8 Apex II 4 K Kappa CCD diffractometer using an exposure time of 30 s/frame. A total of 1318 frames was collected with a frame width of 0.5° covering up to θ=28.00° with 98.3% completeness accomplished.
Geometry. All s.u.'s (except the s.u. in the dihedral angle between two l.s. planes) are estimated using the full covariance matrix. The cell s.u.'s are taken into account individually in the estimation of s.u.'s in distances, angles and torsion angles; correlations between s.u.'s in cell parameters are only used when they are defined by crystal symmetry. An approximate (isotropic) treatment of cell s.u.'s is used for estimating s.u.'s involving l.s. planes.
Refinement. Refinement of *F*^2^ against ALL reflections. The weighted *R*-factor *wR* and goodness of fit *S* are based on *F*^2^, conventional *R*-factors *R* are based on *F*, with *F* set to zero for negative *F*^2^. The threshold expression of *F*^2^ > 2σ(*F*^2^) is used only for calculating *R*-factors(gt) *etc*. and is not relevant to the choice of reflections for refinement. *R*-factors based on *F*^2^ are statistically about twice as large as those based on *F*, and *R*- factors based on ALL data will be even larger.

### Fractional atomic coordinates and isotropic or equivalent isotropic displacement parameters (Å^2^)

**Table:**

	*x*	*y*	*z*	*U*_iso_*/*U*_eq_	Occ. (<1)
Rh1	0	0.5	0.5	0.01620 (12)	
Rh2	0	0	0	0.01681 (12)	
P1	0.17881 (11)	0.45129 (8)	0.59018 (4)	0.0169 (2)	
P2	0.19028 (11)	0.15093 (8)	−0.03218 (4)	0.0169 (2)	
C1	−0.0559 (14)	0.3657 (10)	0.4714 (6)	0.034 (3)	0.5
C2	0.3688 (4)	0.5602 (3)	0.62055 (16)	0.0169 (8)	
C3	0.4759 (4)	0.6069 (3)	0.57726 (17)	0.0196 (8)	
H023	0.4495	0.579	0.5336	0.023*	
C4	0.6194 (4)	0.6927 (3)	0.59619 (18)	0.0210 (8)	
H035	0.6927	0.7215	0.5663	0.025*	
C5	0.6553 (5)	0.7363 (3)	0.65925 (19)	0.0228 (8)	
C6	0.5486 (5)	0.6916 (3)	0.70302 (18)	0.0230 (8)	
H030	0.5727	0.7214	0.7462	0.028*	
C7	0.4080 (4)	0.6042 (3)	0.68391 (17)	0.0198 (8)	
H047	0.3372	0.5738	0.7143	0.024*	
C8	0.8885 (6)	0.8790 (4)	0.6380 (2)	0.0418 (12)	
H04A	0.9481	0.8272	0.6187	0.063*	
H04B	0.9769	0.9412	0.6604	0.063*	
H04C	0.8091	0.9069	0.604	0.063*	
C9	0.2703 (4)	0.3274 (3)	0.58447 (16)	0.0179 (8)	
C10	0.4508 (4)	0.3317 (3)	0.58604 (16)	0.0184 (8)	
H048	0.5295	0.4016	0.5905	0.022*	
C11	0.5171 (5)	0.2356 (3)	0.58120 (17)	0.0221 (8)	
H036	0.64	0.2404	0.5823	0.027*	
C12	0.4043 (5)	0.1327 (3)	0.57470 (17)	0.0220 (8)	
C13	0.2238 (5)	0.1265 (3)	0.57155 (18)	0.0233 (8)	
H022	0.1452	0.0565	0.5662	0.028*	
C14	0.1590 (4)	0.2230 (3)	0.57627 (17)	0.0222 (8)	
H046	0.0355	0.2178	0.5739	0.027*	
C15	0.3688 (6)	−0.0645 (3)	0.5660 (2)	0.0342 (10)	
H05J	0.3069	−0.0728	0.6029	0.051*	
H05K	0.44	−0.1216	0.5657	0.051*	
H05L	0.2822	−0.0724	0.5259	0.051*	
C16	0.0517 (4)	0.4249 (3)	0.65485 (16)	0.0174 (8)	
C17	0.0822 (4)	0.3493 (3)	0.70175 (17)	0.0212 (8)	
H034	0.1791	0.3132	0.7026	0.025*	
C18	−0.0271 (4)	0.3261 (3)	0.74719 (17)	0.0210 (8)	
H033	−0.0038	0.2751	0.7793	0.025*	
C19	−0.1717 (4)	0.3776 (3)	0.74576 (17)	0.0192 (8)	
C20	−0.1985 (4)	0.4567 (3)	0.70120 (17)	0.0190 (8)	
H043	−0.2924	0.4951	0.7016	0.023*	
C21	−0.0880 (4)	0.4794 (3)	0.65610 (17)	0.0197 (8)	
H014	−0.1077	0.5333	0.6254	0.024*	
C22	−0.4399 (4)	0.3816 (4)	0.78430 (18)	0.0257 (9)	
H04D	−0.4143	0.4615	0.7948	0.039*	
H04E	−0.5113	0.3433	0.8143	0.039*	
H04F	−0.5052	0.3664	0.7401	0.039*	
C23	−0.0457 (15)	−0.0644 (11)	−0.0774 (5)	0.020 (2)	0.5
C24	0.0810 (4)	0.2656 (3)	−0.05056 (17)	0.0174 (8)	
C25	0.1519 (4)	0.3536 (3)	−0.08578 (17)	0.0196 (8)	
H037	0.2597	0.353	−0.1006	0.024*	
C26	0.0674 (4)	0.4416 (3)	−0.09933 (17)	0.0184 (8)	
H019	0.1175	0.5006	−0.1232	0.022*	
C27	−0.0899 (4)	0.4437 (3)	−0.07806 (17)	0.0170 (7)	
C28	−0.1621 (4)	0.3576 (3)	−0.04193 (17)	0.0195 (8)	
H044	−0.2683	0.3594	−0.0262	0.023*	
C29	−0.0769 (4)	0.2694 (3)	−0.02936 (17)	0.0172 (8)	
H017	−0.1276	0.2102	−0.0057	0.021*	
C30	−0.1136 (5)	0.6129 (3)	−0.12824 (19)	0.0253 (9)	
H05D	0.0028	0.6508	−0.1055	0.038*	
H05E	−0.1928	0.6659	−0.1347	0.038*	
H05F	−0.102	0.5823	−0.1702	0.038*	
C31	0.2855 (4)	0.1317 (3)	−0.10391 (17)	0.0172 (8)	
C32	0.1737 (4)	0.1092 (3)	−0.16402 (17)	0.0196 (8)	
H021	0.0512	0.1087	−0.1671	0.023*	
C33	0.2403 (5)	0.0879 (3)	−0.21875 (18)	0.0239 (9)	
H026	0.1636	0.0744	−0.2593	0.029*	
C34	0.4202 (5)	0.0860 (3)	−0.21482 (18)	0.0225 (8)	
C35	0.5329 (4)	0.1102 (3)	−0.15605 (17)	0.0207 (8)	
H016	0.6556	0.1114	−0.153	0.025*	
C36	0.4641 (4)	0.1327 (3)	−0.10139 (17)	0.0182 (8)	
H041	0.5418	0.1493	−0.0612	0.022*	
C37	0.6495 (6)	0.0500 (4)	−0.2687 (2)	0.0389 (11)	
H05A	0.727	0.1218	−0.2545	0.058*	
H05B	0.6674	0.0277	−0.3116	0.058*	
H05C	0.6775	−0.0058	−0.2382	0.058*	
C38	0.3798 (4)	0.2067 (3)	0.03055 (17)	0.0178 (8)	
C39	0.4737 (5)	0.1321 (3)	0.06249 (17)	0.0224 (8)	
H031	0.4463	0.0563	0.0479	0.027*	
C40	0.6072 (5)	0.1675 (3)	0.11553 (18)	0.0222 (8)	
H054	0.6728	0.1167	0.136	0.027*	
C41	0.6431 (4)	0.2766 (3)	0.13794 (17)	0.0187 (8)	
C42	0.5510 (4)	0.3518 (3)	0.10570 (18)	0.0194 (8)	
H018	0.5768	0.4274	0.1207	0.023*	
C43	0.4228 (4)	0.3165 (3)	0.05225 (18)	0.0190 (8)	
H032	0.3633	0.3686	0.03	0.023*	
C44	0.8474 (5)	0.2447 (4)	0.22982 (19)	0.0304 (10)	
H05G	0.7578	0.1861	0.2427	0.046*	
H05H	0.922	0.2847	0.2684	0.046*	
H05I	0.9207	0.2117	0.2047	0.046*	
O1	−0.1082 (16)	0.2772 (11)	0.4481 (7)	0.034 (3)	0.5
O2	0.7890 (3)	0.8233 (2)	0.68280 (13)	0.0293 (7)	
O3	0.4817 (3)	0.0430 (2)	0.57075 (13)	0.0283 (6)	
O4	−0.2777 (3)	0.3427 (2)	0.78963 (12)	0.0237 (6)	
O5	−0.085 (2)	−0.1068 (14)	−0.1294 (5)	0.037 (3)	0.5
O6	−0.1854 (3)	0.5247 (2)	−0.09059 (12)	0.0209 (6)	
O7	0.4683 (4)	0.0594 (3)	−0.27137 (13)	0.0319 (7)	
O8	0.7629 (3)	0.3197 (2)	0.19171 (12)	0.0231 (6)	
Cl1	−0.0738 (4)	0.3106 (4)	0.4565 (2)	0.0283 (11)	0.5
Cl2	−0.0659 (5)	−0.0881 (4)	−0.10676 (18)	0.0247 (9)	0.5

### Atomic displacement parameters (Å^2^)

**Table:**

	*U*^11^	*U*^22^	*U*^33^	*U*^12^	*U*^13^	*U*^23^
Rh1	0.01540 (18)	0.0208 (2)	0.0135 (2)	0.00573 (15)	0.00244 (14)	0.00556 (17)
Rh2	0.01738 (18)	0.0173 (2)	0.0175 (2)	0.00511 (15)	0.00556 (15)	0.00546 (17)
P1	0.0156 (4)	0.0228 (6)	0.0141 (4)	0.0068 (4)	0.0039 (3)	0.0060 (4)
P2	0.0165 (4)	0.0174 (5)	0.0181 (5)	0.0053 (4)	0.0043 (3)	0.0059 (4)
C1	0.025 (4)	0.048 (8)	0.023 (4)	0.003 (4)	−0.010 (3)	0.010 (5)
C2	0.0161 (15)	0.021 (2)	0.0156 (17)	0.0075 (14)	0.0034 (13)	0.0050 (15)
C3	0.0186 (16)	0.025 (2)	0.0161 (17)	0.0070 (15)	0.0041 (13)	−0.0002 (16)
C4	0.0189 (16)	0.024 (2)	0.0234 (19)	0.0066 (15)	0.0090 (14)	0.0037 (17)
C5	0.0221 (17)	0.021 (2)	0.027 (2)	0.0076 (15)	0.0029 (15)	0.0021 (17)
C6	0.0269 (18)	0.028 (2)	0.0158 (18)	0.0110 (16)	0.0020 (14)	0.0017 (16)
C7	0.0192 (16)	0.026 (2)	0.0171 (17)	0.0106 (15)	0.0051 (14)	0.0042 (16)
C8	0.034 (2)	0.033 (3)	0.055 (3)	−0.0122 (19)	0.019 (2)	−0.010 (2)
C9	0.0193 (16)	0.023 (2)	0.0124 (16)	0.0049 (14)	0.0043 (13)	0.0066 (15)
C10	0.0205 (16)	0.022 (2)	0.0131 (17)	0.0053 (14)	0.0023 (13)	0.0002 (15)
C11	0.0219 (17)	0.026 (2)	0.0200 (18)	0.0074 (15)	0.0047 (14)	0.0005 (17)
C12	0.0310 (19)	0.023 (2)	0.0154 (17)	0.0126 (16)	0.0066 (15)	0.0015 (16)
C13	0.0254 (17)	0.021 (2)	0.0231 (19)	0.0025 (15)	0.0059 (15)	0.0028 (17)
C14	0.0172 (16)	0.028 (2)	0.0224 (19)	0.0049 (15)	0.0058 (14)	0.0052 (17)
C15	0.044 (2)	0.020 (2)	0.043 (3)	0.0120 (19)	0.011 (2)	0.005 (2)
C16	0.0170 (15)	0.022 (2)	0.0160 (17)	0.0080 (14)	0.0059 (13)	0.0054 (15)
C17	0.0199 (16)	0.027 (2)	0.0188 (18)	0.0092 (15)	0.0023 (14)	0.0082 (17)
C18	0.0200 (16)	0.028 (2)	0.0177 (18)	0.0097 (15)	0.0030 (14)	0.0107 (16)
C19	0.0170 (15)	0.027 (2)	0.0153 (17)	0.0050 (15)	0.0060 (13)	0.0036 (16)
C20	0.0174 (15)	0.025 (2)	0.0181 (18)	0.0102 (15)	0.0060 (13)	0.0041 (16)
C21	0.0191 (16)	0.024 (2)	0.0175 (18)	0.0065 (15)	0.0034 (13)	0.0071 (16)
C22	0.0178 (16)	0.038 (3)	0.025 (2)	0.0091 (16)	0.0084 (15)	0.0062 (19)
C23	0.018 (4)	0.021 (6)	0.020 (6)	0.002 (4)	0.008 (5)	0.005 (5)
C24	0.0195 (16)	0.016 (2)	0.0179 (17)	0.0078 (14)	0.0021 (13)	0.0012 (15)
C25	0.0163 (15)	0.023 (2)	0.0228 (19)	0.0068 (14)	0.0086 (13)	0.0047 (16)
C26	0.0185 (15)	0.018 (2)	0.0209 (18)	0.0051 (14)	0.0075 (14)	0.0059 (16)
C27	0.0197 (16)	0.014 (2)	0.0195 (18)	0.0072 (14)	0.0046 (13)	0.0001 (15)
C28	0.0168 (15)	0.024 (2)	0.0215 (18)	0.0076 (14)	0.0088 (14)	0.0045 (16)
C29	0.0173 (15)	0.016 (2)	0.0200 (18)	0.0047 (14)	0.0055 (13)	0.0062 (15)
C30	0.0275 (18)	0.025 (2)	0.028 (2)	0.0104 (16)	0.0100 (16)	0.0112 (18)
C31	0.0203 (16)	0.0129 (19)	0.0197 (18)	0.0057 (14)	0.0037 (14)	0.0060 (15)
C32	0.0174 (15)	0.020 (2)	0.0217 (19)	0.0059 (14)	0.0023 (14)	0.0052 (16)
C33	0.0270 (18)	0.029 (2)	0.0160 (18)	0.0091 (16)	−0.0015 (14)	0.0035 (17)
C34	0.0291 (18)	0.022 (2)	0.0185 (18)	0.0061 (16)	0.0088 (15)	0.0024 (16)
C35	0.0207 (16)	0.023 (2)	0.0202 (19)	0.0075 (15)	0.0062 (14)	0.0030 (16)
C36	0.0194 (15)	0.018 (2)	0.0187 (18)	0.0058 (14)	0.0042 (13)	0.0060 (15)
C37	0.037 (2)	0.057 (3)	0.030 (2)	0.018 (2)	0.0184 (19)	−0.001 (2)
C38	0.0184 (15)	0.019 (2)	0.0161 (17)	0.0047 (14)	0.0032 (13)	0.0053 (15)
C39	0.0273 (18)	0.022 (2)	0.0194 (19)	0.0087 (16)	0.0037 (15)	0.0006 (16)
C40	0.0239 (17)	0.025 (2)	0.0198 (18)	0.0098 (16)	0.0029 (14)	0.0068 (17)
C41	0.0150 (15)	0.027 (2)	0.0165 (17)	0.0059 (14)	0.0070 (13)	0.0026 (16)
C42	0.0183 (15)	0.017 (2)	0.0246 (19)	0.0038 (14)	0.0081 (14)	0.0014 (16)
C43	0.0130 (14)	0.021 (2)	0.0252 (19)	0.0060 (14)	0.0070 (13)	0.0058 (16)
C44	0.031 (2)	0.038 (3)	0.022 (2)	0.0116 (18)	−0.0023 (16)	0.0038 (19)
O1	0.025 (4)	0.048 (8)	0.023 (4)	0.003 (4)	−0.010 (3)	0.010 (5)
O2	0.0238 (13)	0.0297 (18)	0.0314 (16)	0.0005 (12)	0.0029 (11)	−0.0069 (13)
O3	0.0341 (14)	0.0233 (17)	0.0329 (16)	0.0137 (12)	0.0114 (12)	0.0018 (13)
O4	0.0215 (12)	0.0350 (17)	0.0199 (13)	0.0121 (11)	0.0102 (10)	0.0124 (12)
O5	0.050 (6)	0.043 (7)	0.019 (7)	−0.001 (4)	0.012 (5)	0.004 (6)
O6	0.0208 (12)	0.0209 (15)	0.0264 (14)	0.0120 (10)	0.0095 (10)	0.0099 (12)
O7	0.0364 (15)	0.045 (2)	0.0190 (14)	0.0174 (14)	0.0089 (12)	0.0006 (13)
O8	0.0228 (12)	0.0276 (17)	0.0188 (13)	0.0068 (11)	0.0011 (10)	0.0009 (12)
Cl1	0.0235 (17)	0.031 (3)	0.026 (2)	0.0019 (17)	−0.0072 (14)	0.002 (2)
Cl2	0.0301 (15)	0.026 (2)	0.017 (3)	0.0001 (14)	0.007 (2)	0.004 (2)

### Geometric parameters (Å, °)

**Table:**

Rh1—C1	1.699 (12)	C19—C20	1.385 (5)
Rh1—C1^i^	1.699 (13)	C20—C21	1.385 (5)
Rh1—P1^i^	2.3257 (10)	C20—H043	0.95
Rh1—P1	2.3257 (10)	C21—H014	0.95
Rh1—Cl1	2.416 (5)	C22—O4	1.431 (4)
Rh1—Cl1^i^	2.416 (5)	C22—H04D	0.98
Rh2—C23	1.751 (11)	C22—H04E	0.98
Rh2—C23^ii^	1.751 (11)	C22—H04F	0.98
Rh2—P2^ii^	2.3321 (11)	C23—Cl2	0.660 (9)
Rh2—P2	2.3321 (11)	C23—O5	1.171 (13)
Rh2—Cl2	2.410 (4)	C24—C29	1.392 (5)
Rh2—Cl2^ii^	2.410 (4)	C24—C25	1.402 (5)
P1—C16	1.815 (3)	C25—C26	1.386 (5)
P1—C2	1.817 (4)	C25—H037	0.95
P1—C9	1.817 (4)	C26—C27	1.384 (5)
P2—C24	1.804 (4)	C26—H019	0.95
P2—C31	1.824 (4)	C27—O6	1.363 (4)
P2—C38	1.827 (4)	C27—C28	1.401 (5)
C1—Cl1	0.720 (12)	C28—C29	1.388 (5)
C1—O1	1.157 (18)	C28—H044	0.95
C2—C7	1.393 (5)	C29—H017	0.95
C2—C3	1.397 (5)	C30—O6	1.438 (4)
C3—C4	1.383 (5)	C30—H05D	0.98
C3—H023	0.95	C30—H05E	0.98
C4—C5	1.387 (5)	C30—H05F	0.98
C4—H035	0.95	C31—C36	1.389 (5)
C5—O2	1.365 (5)	C31—C32	1.404 (5)
C5—C6	1.396 (5)	C32—C33	1.383 (5)
C6—C7	1.382 (5)	C32—H021	0.95
C6—H030	0.95	C33—C34	1.403 (5)
C7—H047	0.95	C33—H026	0.95
C8—O2	1.424 (5)	C34—O7	1.361 (5)
C8—H04A	0.98	C34—C35	1.386 (5)
C8—H04B	0.98	C35—C36	1.395 (5)
C8—H04C	0.98	C35—H016	0.95
C9—C14	1.397 (5)	C36—H041	0.95
C9—C10	1.400 (5)	C37—O7	1.438 (5)
C10—C11	1.391 (5)	C37—H05A	0.98
C10—H048	0.95	C37—H05B	0.98
C11—C12	1.386 (5)	C37—H05C	0.98
C11—H036	0.95	C38—C43	1.379 (5)
C12—O3	1.367 (5)	C38—C39	1.399 (5)
C12—C13	1.392 (5)	C39—C40	1.396 (5)
C13—C14	1.388 (6)	C39—H031	0.95
C13—H022	0.95	C40—C41	1.376 (6)
C14—H046	0.95	C40—H054	0.95
C15—O3	1.433 (5)	C41—O8	1.370 (4)
C15—H05J	0.98	C41—C42	1.399 (5)
C15—H05K	0.98	C42—C43	1.378 (5)
C15—H05L	0.98	C42—H018	0.95
C16—C17	1.393 (5)	C43—H032	0.95
C16—C21	1.394 (5)	C44—O8	1.422 (4)
C17—C18	1.385 (5)	C44—H05G	0.98
C17—H034	0.95	C44—H05H	0.98
C18—C19	1.399 (5)	C44—H05I	0.98
C18—H033	0.95	O5—Cl2	0.511 (10)
C19—O4	1.363 (4)		
			
C1—Rh1—C1^i^	180.000 (4)	C17—C18—H033	120
C1—Rh1—P1^i^	89.5 (4)	C19—C18—H033	120
C1^i^—Rh1—P1^i^	90.5 (4)	O4—C19—C20	125.3 (3)
C1—Rh1—P1	90.5 (4)	O4—C19—C18	115.1 (3)
C1^i^—Rh1—P1	89.5 (4)	C20—C19—C18	119.6 (3)
P1^i^—Rh1—P1	180	C19—C20—C21	119.7 (3)
C1—Rh1—Cl1	1.9 (4)	C19—C20—H043	120.1
C1^i^—Rh1—Cl1	178.1 (4)	C21—C20—H043	120.1
P1^i^—Rh1—Cl1	88.80 (9)	C20—C21—C16	121.3 (3)
P1—Rh1—Cl1	91.20 (9)	C20—C21—H014	119.3
C1—Rh1—Cl1^i^	178.1 (4)	C16—C21—H014	119.3
C1^i^—Rh1—Cl1^i^	1.9 (4)	O4—C22—H04D	109.5
P1^i^—Rh1—Cl1^i^	91.20 (9)	O4—C22—H04E	109.5
P1—Rh1—Cl1^i^	88.80 (9)	H04D—C22—H04E	109.5
Cl1—Rh1—Cl1^i^	180.0000 (10)	O4—C22—H04F	109.5
C23—Rh2—C23^ii^	180.0 (9)	H04D—C22—H04F	109.5
C23—Rh2—P2^ii^	88.1 (4)	H04E—C22—H04F	109.5
C23^ii^—Rh2—P2^ii^	91.9 (4)	Cl2—C23—Rh2	177.8 (15)
C23—Rh2—P2	91.9 (4)	O5—C23—Rh2	176.8 (16)
C23^ii^—Rh2—P2	88.1 (4)	C29—C24—C25	117.9 (3)
P2^ii^—Rh2—P2	180.00 (5)	C29—C24—P2	120.5 (3)
C23—Rh2—Cl2	0.6 (4)	C25—C24—P2	121.6 (3)
C23^ii^—Rh2—Cl2	179.4 (4)	C26—C25—C24	121.2 (3)
P2^ii^—Rh2—Cl2	87.94 (10)	C26—C25—H037	119.4
P2—Rh2—Cl2	92.06 (10)	C24—C25—H037	119.4
C23—Rh2—Cl2^ii^	179.4 (4)	C27—C26—C25	120.1 (3)
C23^ii^—Rh2—Cl2^ii^	0.6 (4)	C27—C26—H019	120
P2^ii^—Rh2—Cl2^ii^	92.06 (10)	C25—C26—H019	120
P2—Rh2—Cl2^ii^	87.94 (10)	O6—C27—C26	124.4 (3)
Cl2—Rh2—Cl2^ii^	180.0 (2)	O6—C27—C28	115.7 (3)
C16—P1—C2	106.79 (17)	C26—C27—C28	119.8 (3)
C16—P1—C9	103.67 (16)	C29—C28—C27	119.4 (3)
C2—P1—C9	104.54 (16)	C29—C28—H044	120.3
C16—P1—Rh1	109.09 (11)	C27—C28—H044	120.3
C2—P1—Rh1	112.92 (11)	C28—C29—C24	121.6 (3)
C9—P1—Rh1	118.87 (12)	C28—C29—H017	119.2
C24—P2—C31	103.44 (16)	C24—C29—H017	119.2
C24—P2—C38	104.95 (17)	O6—C30—H05D	109.5
C31—P2—C38	104.75 (16)	O6—C30—H05E	109.5
C24—P2—Rh2	111.46 (12)	H05D—C30—H05E	109.5
C31—P2—Rh2	118.04 (12)	O6—C30—H05F	109.5
C38—P2—Rh2	112.97 (11)	H05D—C30—H05F	109.5
Cl1—C1—Rh1	173.6 (14)	H05E—C30—H05F	109.5
O1—C1—Rh1	173.2 (14)	C36—C31—C32	118.0 (3)
C7—C2—C3	118.0 (3)	C36—C31—P2	122.7 (3)
C7—C2—P1	123.5 (3)	C32—C31—P2	119.2 (3)
C3—C2—P1	118.3 (3)	C33—C32—C31	120.6 (3)
C4—C3—C2	121.8 (3)	C33—C32—H021	119.7
C4—C3—H023	119.1	C31—C32—H021	119.7
C2—C3—H023	119.1	C32—C33—C34	120.5 (3)
C3—C4—C5	119.4 (3)	C32—C33—H026	119.8
C3—C4—H035	120.3	C34—C33—H026	119.8
C5—C4—H035	120.3	O7—C34—C35	125.4 (3)
O2—C5—C4	124.7 (3)	O7—C34—C33	115.0 (3)
O2—C5—C6	115.7 (4)	C35—C34—C33	119.6 (3)
C4—C5—C6	119.6 (4)	C34—C35—C36	119.2 (3)
C7—C6—C5	120.4 (4)	C34—C35—H016	120.4
C7—C6—H030	119.8	C36—C35—H016	120.4
C5—C6—H030	119.8	C31—C36—C35	122.0 (3)
C6—C7—C2	120.7 (3)	C31—C36—H041	119
C6—C7—H047	119.6	C35—C36—H041	119
C2—C7—H047	119.6	O7—C37—H05A	109.5
O2—C8—H04A	109.5	O7—C37—H05B	109.5
O2—C8—H04B	109.5	H05A—C37—H05B	109.5
H04A—C8—H04B	109.5	O7—C37—H05C	109.5
O2—C8—H04C	109.5	H05A—C37—H05C	109.5
H04A—C8—H04C	109.5	H05B—C37—H05C	109.5
H04B—C8—H04C	109.5	C43—C38—C39	118.6 (3)
C14—C9—C10	117.3 (3)	C43—C38—P2	123.0 (3)
C14—C9—P1	120.2 (3)	C39—C38—P2	118.1 (3)
C10—C9—P1	122.5 (3)	C40—C39—C38	120.9 (4)
C11—C10—C9	121.3 (4)	C40—C39—H031	119.5
C11—C10—H048	119.4	C38—C39—H031	119.5
C9—C10—H048	119.4	C41—C40—C39	119.5 (3)
C12—C11—C10	120.2 (3)	C41—C40—H054	120.2
C12—C11—H036	119.9	C39—C40—H054	120.2
C10—C11—H036	119.9	O8—C41—C40	124.9 (3)
O3—C12—C11	116.0 (3)	O8—C41—C42	115.3 (4)
O3—C12—C13	124.4 (4)	C40—C41—C42	119.7 (3)
C11—C12—C13	119.5 (4)	C43—C42—C41	120.2 (4)
C14—C13—C12	119.8 (4)	C43—C42—H018	119.9
C14—C13—H022	120.1	C41—C42—H018	119.9
C12—C13—H022	120.1	C42—C43—C38	121.0 (3)
C13—C14—C9	121.9 (3)	C42—C43—H032	119.5
C13—C14—H046	119.1	C38—C43—H032	119.5
C9—C14—H046	119.1	O8—C44—H05G	109.5
O3—C15—H05J	109.5	O8—C44—H05H	109.5
O3—C15—H05K	109.5	H05G—C44—H05H	109.5
H05J—C15—H05K	109.5	O8—C44—H05I	109.5
O3—C15—H05L	109.5	H05G—C44—H05I	109.5
H05J—C15—H05L	109.5	H05H—C44—H05I	109.5
H05K—C15—H05L	109.5	C5—O2—C8	117.3 (3)
C17—C16—C21	118.3 (3)	C12—O3—C15	117.2 (3)
C17—C16—P1	123.1 (3)	C19—O4—C22	117.1 (3)
C21—C16—P1	118.5 (2)	C27—O6—C30	116.2 (3)
C18—C17—C16	120.8 (3)	C34—O7—C37	116.8 (3)
C18—C17—H034	119.6	C41—O8—C44	117.6 (3)
C16—C17—H034	119.6	O5—Cl2—C23	177 (2)
C17—C18—C19	120.0 (3)	O5—Cl2—Rh2	176 (2)
			
C1—Rh1—P1—C16	−93.0 (4)	C18—C19—C20—C21	3.7 (6)
C1^i^—Rh1—P1—C16	87.0 (4)	C19—C20—C21—C16	−0.6 (6)
Cl1—Rh1—P1—C16	−94.75 (17)	C17—C16—C21—C20	−2.3 (6)
Cl1^i^—Rh1—P1—C16	85.25 (17)	P1—C16—C21—C20	175.0 (3)
C1—Rh1—P1—C2	148.5 (4)	C31—P2—C24—C29	144.7 (3)
C1^i^—Rh1—P1—C2	−31.5 (4)	C38—P2—C24—C29	−105.8 (3)
Cl1—Rh1—P1—C2	146.68 (16)	Rh2—P2—C24—C29	16.9 (3)
Cl1^i^—Rh1—P1—C2	−33.32 (16)	C31—P2—C24—C25	−35.9 (3)
C1—Rh1—P1—C9	25.5 (4)	C38—P2—C24—C25	73.6 (3)
C1^i^—Rh1—P1—C9	−154.5 (4)	Rh2—P2—C24—C25	−163.7 (3)
Cl1—Rh1—P1—C9	23.70 (16)	C29—C24—C25—C26	−0.2 (5)
Cl1^i^—Rh1—P1—C9	−156.30 (16)	P2—C24—C25—C26	−179.6 (3)
C23—Rh2—P2—C24	97.8 (4)	C24—C25—C26—C27	−0.1 (6)
C23^ii^—Rh2—P2—C24	−82.2 (4)	C25—C26—C27—O6	−177.9 (3)
Cl2—Rh2—P2—C24	97.21 (17)	C25—C26—C27—C28	1.0 (6)
Cl2^ii^—Rh2—P2—C24	−82.79 (17)	O6—C27—C28—C29	177.3 (3)
C23—Rh2—P2—C31	−21.7 (4)	C26—C27—C28—C29	−1.7 (6)
C23^ii^—Rh2—P2—C31	158.3 (4)	C27—C28—C29—C24	1.5 (6)
Cl2—Rh2—P2—C31	−22.28 (16)	C25—C24—C29—C28	−0.5 (5)
Cl2^ii^—Rh2—P2—C31	157.72 (16)	P2—C24—C29—C28	178.9 (3)
C23—Rh2—P2—C38	−144.3 (4)	C24—P2—C31—C36	124.8 (3)
C23^ii^—Rh2—P2—C38	35.7 (4)	C38—P2—C31—C36	15.1 (3)
Cl2—Rh2—P2—C38	−144.90 (17)	Rh2—P2—C31—C36	−111.6 (3)
Cl2^ii^—Rh2—P2—C38	35.10 (17)	C24—P2—C31—C32	−58.5 (3)
C16—P1—C2—C7	4.8 (3)	C38—P2—C31—C32	−168.2 (3)
C9—P1—C2—C7	−104.6 (3)	Rh2—P2—C31—C32	65.1 (3)
Rh1—P1—C2—C7	124.7 (3)	C36—C31—C32—C33	0.5 (5)
C16—P1—C2—C3	−171.4 (3)	P2—C31—C32—C33	−176.2 (3)
C9—P1—C2—C3	79.1 (3)	C31—C32—C33—C34	1.4 (6)
Rh1—P1—C2—C3	−51.5 (3)	C32—C33—C34—O7	177.3 (3)
C7—C2—C3—C4	1.4 (5)	C32—C33—C34—C35	−2.6 (6)
P1—C2—C3—C4	177.8 (3)	O7—C34—C35—C36	−178.0 (3)
C2—C3—C4—C5	−2.1 (6)	C33—C34—C35—C36	1.9 (6)
C3—C4—C5—O2	−177.6 (3)	C32—C31—C36—C35	−1.2 (5)
C3—C4—C5—C6	1.4 (6)	P2—C31—C36—C35	175.4 (3)
O2—C5—C6—C7	179.2 (3)	C34—C35—C36—C31	0.0 (6)
C4—C5—C6—C7	0.1 (6)	C24—P2—C38—C43	−3.7 (3)
C5—C6—C7—C2	−0.9 (5)	C31—P2—C38—C43	104.9 (3)
C3—C2—C7—C6	0.1 (5)	Rh2—P2—C38—C43	−125.3 (3)
P1—C2—C7—C6	−176.1 (3)	C24—P2—C38—C39	169.4 (3)
C16—P1—C9—C14	54.2 (3)	C31—P2—C38—C39	−82.0 (3)
C2—P1—C9—C14	165.9 (3)	Rh2—P2—C38—C39	47.8 (3)
Rh1—P1—C9—C14	−67.0 (3)	C43—C38—C39—C40	0.5 (5)
C16—P1—C9—C10	−127.8 (3)	P2—C38—C39—C40	−172.9 (3)
C2—P1—C9—C10	−16.0 (3)	C38—C39—C40—C41	2.1 (5)
Rh1—P1—C9—C10	111.0 (3)	C39—C40—C41—O8	175.8 (3)
C14—C9—C10—C11	−1.5 (5)	C39—C40—C41—C42	−2.8 (5)
P1—C9—C10—C11	−179.6 (3)	O8—C41—C42—C43	−177.8 (3)
C9—C10—C11—C12	0.0 (5)	C40—C41—C42—C43	1.0 (5)
C10—C11—C12—O3	−179.6 (3)	C41—C42—C43—C38	1.7 (5)
C10—C11—C12—C13	1.5 (5)	C39—C38—C43—C42	−2.4 (5)
O3—C12—C13—C14	179.8 (3)	P2—C38—C43—C42	170.6 (3)
C11—C12—C13—C14	−1.3 (5)	C4—C5—O2—C8	5.7 (6)
C12—C13—C14—C9	−0.3 (6)	C6—C5—O2—C8	−173.3 (4)
C10—C9—C14—C13	1.7 (5)	C11—C12—O3—C15	178.2 (3)
P1—C9—C14—C13	179.8 (3)	C13—C12—O3—C15	−2.9 (5)
C2—P1—C16—C17	−88.3 (4)	C20—C19—O4—C22	7.5 (6)
C9—P1—C16—C17	21.8 (4)	C18—C19—O4—C22	−171.7 (3)
Rh1—P1—C16—C17	149.4 (3)	C26—C27—O6—C30	−0.4 (5)
C2—P1—C16—C21	94.6 (3)	C28—C27—O6—C30	−179.3 (3)
C9—P1—C16—C21	−155.4 (3)	C35—C34—O7—C37	3.5 (6)
Rh1—P1—C16—C21	−27.8 (3)	C33—C34—O7—C37	−176.4 (3)
C21—C16—C17—C18	2.1 (6)	C40—C41—O8—C44	−4.7 (5)
P1—C16—C17—C18	−175.1 (3)	C42—C41—O8—C44	173.9 (3)
C16—C17—C18—C19	1.0 (6)	O1—C1—Cl1—Rh1	132 (13)
C17—C18—C19—O4	175.4 (4)	P1^i^—Rh1—Cl1—C1	−110 (11)
C17—C18—C19—C20	−3.9 (6)	P1—Rh1—Cl1—C1	70 (11)
O4—C19—C20—C21	−175.5 (4)		

Symmetry codes: (i) −*x*, −*y*+1, −*z*+1; (ii) −*x*, −*y*, −*z*.

### Hydrogen-bond geometry (Å, °)

**Table:**

*D*—H···*A*	*D*—H	H···*A*	*D*···*A*	*D*—H···*A*
C14—H046···Cl1	0.95	2.81	3.191 (5)	105.
C32—H021···Cl2	0.95	2.82	3.157 (5)	102.
C8—H04A···Cl1^iii^	0.98	2.73	3.693 (7)	169.
C8—H04A···O1^iii^	0.98	2.52	3.486 (18)	168.

Symmetry codes: (iii) −*x*+1, −*y*+1, −*z*+1.
